# Advances of Nanozyme-Driven Multimodal Sensing Strategies in Point-of-Care Testing

**DOI:** 10.3390/bios15060375

**Published:** 2025-06-10

**Authors:** Ziyi Chang, Qingjie Fu, Mengke Wang, Demin Duan

**Affiliations:** 1 Graduate School of Xinxiang Medical University, Xinxiang 453003, China; changziyi@nanozyme.tech; 2 Nanozyme Laboratory in Zhongyuan, Henan Academy of Innovations in Medical Science, Zhengzhou 451163, China; fuqingjie@nanozyme.tech; 3 CAS Engineering Laboratory for Nanozyme, National Laboratory of Biomacromolecules, Institute of Biophysics, Chinese Academy of Sciences, Beijing 100101, China

**Keywords:** nanozymes, POCT, sensing strategies, biomarker detection

## Abstract

Point-of-care testing (POCT) has garnered widespread attention due to its rapid, convenient, and efficient detection capabilities, particularly playing an increasingly pivotal role in medical diagnostics and significantly improving the efficiency and quality of healthcare services. Nanozymes, as novel enzyme-mimicking materials, have emerged as a research hotspot owing to their superior catalytic performance, low cost, and robust stability. This review provides a systematic overview of the fundamental characteristics and classifications of nanozymes, along with various sensing strategies employed in POCT applications, colorimetric, electrochemical, fluorescent, chemiluminescent, and surface-enhanced Raman scattering (SERS)-based approaches. Furthermore, this review highlights innovative designs that enhance the sensitivity and accuracy of POCT across multiple domains, such as biomarker detection, environmental monitoring, and food safety analysis, thereby offering novel perspectives for the practical implementation of nanozymes in point-of-care diagnostics. Finally, this review analyzes current challenges in nanozyme-based POCT systems, including limitations in optimizing catalytic activity, ensuring nanozyme homogeneity, and achieving large-scale production, while proposing future development trajectories.

## 1. Introduction

Laboratory analysis serves as the cornerstone of scientific research, providing precise and reliable data for the field of testing. However, traditional laboratory methods often fail to meet the demands for rapid on-site analysis. With the continuous advancement of science and technology, point-of-care testing (POCT) has emerged. POCT refers to rapid and simple medical testing performed near the patient, necessitating miniaturized instruments that are portable, flexible, fast, and accurate [1]. As a rapidly developing technology in both analytical testing and clinical medicine, POCT offers advantages such as simplified testing procedures, independence from specific locations, elimination of the need for bulky precision instruments, operation without specialized personnel, and the ability to generate accurate reports in a short time [2,3].

POCT primarily relies on biosensing systems combined with information technology for data readout [4,5]; the sensor elements employed play a pivotal role in providing essential medical monitoring data and ensuring that patients receive timely, critical care. By offering rapid, convenient, and accurate on-site results, POCT has significantly enhanced the efficiency and quality of healthcare services, thereby improving patient care, reducing medical costs, strengthening disease management, and addressing public health crises. Among these, immunochromatographic techniques, as one of the most commonly employed rapid detection methods in the POCT domain, are mainly used for qualitative analyses, though they exhibit limitations in sensitivity and quantitative analysis [6]. With the advent of miniaturized and integrated microfluidic chip technology (microfluidic chip) and next-generation biosensors, both the sensitivity and specificity of detection have been substantially improved, heralding a revolutionary breakthrough in the POCT field [7,8]. Recent years have witnessed the integration of smart devices and intelligent apps with POCT, along with groundbreaking innovations in wearable instrumentation. This convergence has driven a technological leap in POCT from qualitative to quantitative analysis [9]. Today, POCT is experiencing comprehensive development across diverse fields such as medicine, agriculture, food processing, and environmental management, emerging as a cutting-edge precision detection technology. Although POCT has significantly reduced testing times, the ever-growing demand for enhanced sensitivity has rendered traditional approaches insufficient for modern laboratory requirements. For instance, in scenarios involving the detection of early-stage lesions, low-abundance pathogens, or the analysis of complex samples, limited sensitivity remains a critical challenge. This issue is particularly critical in the face of highly fatal, rapidly emerging acute infectious diseases, where there is an urgent need for point-of-care products that offer both high specificity and high sensitivity.

Nanozymes, as an emerging class of nanomaterials, combine the catalytic activity of enzymes with the unique physicochemical properties of nanoscale substances [10]. In 2007, the team led by Chinese scientist Yan Xiyun reported that iron oxide nanoparticles (Fe_3_O_4_ NPs) exhibit catalytic activity similar to that of horseradish peroxidase (HRP) [11,12]. This discovery not only laid the foundation for nanozyme research and applications, but also challenged the prevailing notion that nanomaterials are inert. Furthermore, compared to natural enzymes, nanozymes offer several advantages, including tunable catalytic activity, low production cost, high stability, and ease of large-scale manufacturing, making them promising substitutes for natural enzymes [13,14]. With ongoing research and development in recent years, nanozymes have found widespread applications in disease diagnosis and treatment, biomedicine, environmental remediation, and agriculture-related fields [15,16,17,18,19]. Recently, various types of nanozymes have been incorporated into the development of biosensing systems, where their unique enzyme-mimetic activity and cost-effectiveness have drawn significant attention, particularly in the realm of POCT. Moreover, by leveraging the inherent functionalities of nanozymes for targeted modifications, novel signal amplification strategies have been devised, opening new avenues to enhance the sensitivity and accuracy of POCT. As nanotechnology continues to advance, the scope and impact of POCT are expected to expand further. Currently, several comprehensive reviews on nanozyme-based POCT have been published. One such review by Professor Cao and colleagues examines the application of nanozymes in POCT, including infectious disease detection, chronic disease monitoring, and environmental pollutant assessment, while discussing their promising prospects in biosensor development. The review further highlights the unique advantages of nanozymes in POCT applications, notably, their high sensitivity, low cost, rapid response, and superior selectivity [20]. Zhang et al. reported a comprehensive review of the latest advancements in nanozyme-based sensors for rapid detection [21]. Their work introduced various detection methods utilizing different signal readouts, and spanned multiple aspects from the underlying principles to specific applications. Wang et al. provided a concise overview of commonly used POCT devices for environmental pollutant detection, detailing methods to identify toxic ions, phenolic compounds, pesticide residues, antibiotic residues, and pathogens [22]. These reviews predominantly classify nanozyme applications by their catalytic activity or emphasize their unique roles in specific POCT fields such as environmental monitoring and clinical diagnostics.

**Figure 1:**
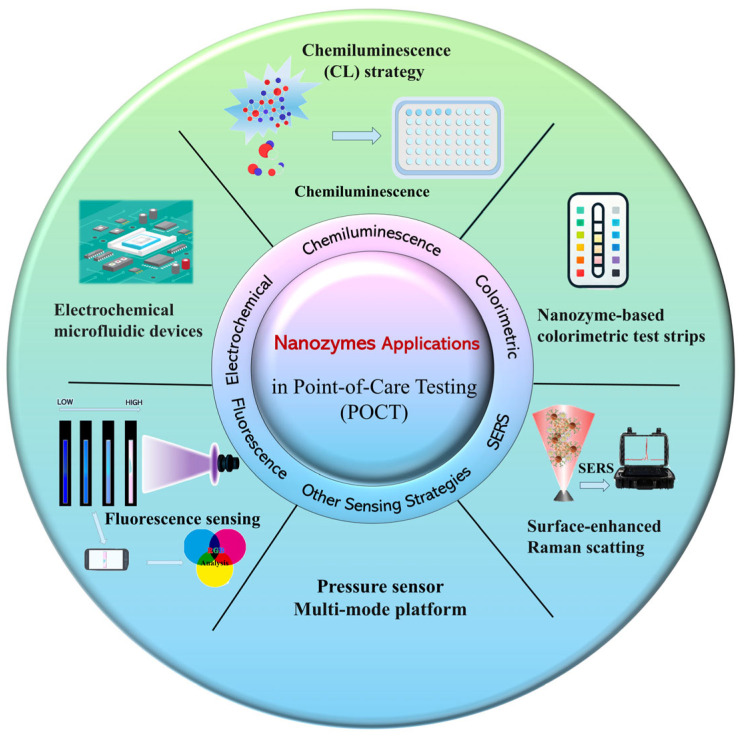
The sensing strategies of nanozymes in POCT applications.

In this review, we aim to further explore recent advancements in novel sensing strategies and performance regulation of nanozymes. Focusing on interdisciplinary multimodal sensing strategies, we comprehensively examine the applications of nanozymes across various sensing platforms. This work systematically summarizes the mechanisms of nanozyme-based POCT in colorimetric, electrochemical, fluorescent, chemiluminescent, and surface-enhanced Raman scattering (SERS) sensing methodologies, along with their corresponding practical applications. Finally, we provide some insights and discussions regarding current challenges, potential solutions, and future perspectives for nanozyme implementation in POCT applications. This review is expected to offer new research directions and valuable references for scholars in this field (Figure 1).

## 2. Classification and Activity Regulation of Nanozymes for POCT

Since nanozymes were first reported to exhibit enzyme-like catalytic activity, people’s understanding and exploration of these materials have advanced considerably. Research has demonstrated that the classifications of nanozymes are diversified according to their core catalytic activities as well as catalytic mechanisms. Leveraging their distinctive structural characteristics and high tunability, targeted synthesis and modification via various physical and chemical methods can markedly enhance their catalytic efficiency and selectivity [23]. Meanwhile, a comprehensive investigation into the catalytic mechanism, diverse classification, and regulatory strategies of nanozymes holds the potential to facilitate a comprehensive exploration of their enzyme-like activities, providing a theoretical foundation for the future innovation of nanozymes and the development of novel nanozymes. In the following sections, we present a comprehensive analysis and discussion of the various classifications of nanozymes, along with the strategies employed for their synthesis and modification.

### 2.1. Classification of Nanozymes

Natural enzymes, synthesized within living organisms, are highly efficient biocatalysts that are highly prized in various catalytic fields due to their exceptional substrate specificity and selectivity. However, their broader application is hampered by high production costs, sensitivity to environmental conditions (such as temperature, pressure, and pH), and challenges associated with storage and recovery, all of which indirectly affect their catalytic performance and stability, thereby limiting their broader application. The advent of nanozymes has effectively remedied these deficiencies [24]. Nanozymes are a class of nanomaterials that exhibit catalytic activity similar to that of natural enzymes [25]. This definition enables us to classify nanozymes based on both their enzyme-like activities and their intrinsic structures. From the perspective of catalytic activity, natural enzymes exhibit a diverse range of catalytic types; however, the nanozymes identified thus far do not encompass the full spectrum of activities found in natural enzymes. Instead, the enzyme-like activities of nanozymes can be primarily categorized into six types: oxidoreductase-like, hydrolase-like, lyase-like, transferase-like, ligase-like, and isomerase-like. Among these, the oxidoreductase-like nanozymes have been most extensively reported, and they can be further subdivided into oxidases (OXDs), peroxidases (PODs), catalases (CATs), and superoxide dismutases (SODs) [26,27,28,29]. This classification framework facilitates an in-depth understanding of the catalytic mechanisms of nanozymes and extends the investigation of their enzyme-mimicking activities to a broad range of applications. Classifying nanozymes based on enzyme-like activity is currently the most widely used and common approach. To achieve a more comprehensive, multidimensional understanding of nanozymes, they can also be categorized according to differences in their core structures into the following major classes: metal-based nanozymes, metal oxide-based nanozymes, metal–organic frameworks (MOFs), and single-atom nanozymes, as well as carbon-based nanozymes [30]. This classification highlights the diversity in nanozyme design and their potential for tailored applications (Table 1).

Metal-based nanozymes are nanoscale materials composed of metals or their derivatives that exhibit enzyme-mimicking activities, with embedded metal nanoparticles acting as catalytic active centers [31]. This property enables them to demonstrate superior catalytic efficiency and stability compared to non-metal nanozymes. Reported metal nanomaterial-based nanozymes can be broadly classified by their structural characteristics into monometallic [32], metal alloys [33,34,35], metal oxides [36], metal core/shell nanostructures [37], and hybrid nanomaterials [38]. Due to their unique geometric and electronic structures as well as corrosion resistance, metal and metal oxide nanomaterials have garnered extensive attention; their excellent electronic properties contribute to remarkable performance in applications such as electrocatalysis, biosensing, and fuel cells [39]. However, so-called monometallic nanomaterials (e.g., Au, Ag, Pt), i.e., noble metal nanozymes, tend to aggregate into nanoclusters, reducing catalytic performance, and most are biotoxic, which limits their applications [40]. In contrast, metal oxide nanozymes offer significant advantages due to their lower cost, straightforward synthesis, and non-toxic nature. Their inherent surface oxide layer minimizes the release of metal ions [41]. This superior antioxidant property makes metal oxide nanozymes a leader in mimicking the activity of antioxidant enzymes (i.e., SOD, CAT, and GSH-Px) in the first-line defence mechanism [42]. For example, CeO_2_ nanoparticles have been reported to have antioxidant properties that can be used to prevent oxidative stress, inflammation, neuroprotection, and bacteriostasis, among others [43,44,45,46]. Metal oxide nanomaterials also have many unique properties, such as catalytic, magnetic, UV-absorbing, and dielectric properties [47]. Among them, magnetic iron oxide nanoenzymes (IONzyme, including Fe_3_O_4_ and Fe_2_O_3_) are the most classical and the earliest nanozymes to be discovered [48].

Compared to other metal and metal oxide nanozymes, metal–organic frameworks (MOFs) and single-atom nanozymes (SANAs) have recently attracted considerable attention from researchers. MOFs are typically porous, network-like crystalline materials formed through the self-assembly of organic ligands with metal ions or metal clusters (usually transition metal ions) via coordination interactions [49,50,51]. Owing to their abundant active sites, high specific surface area, high porosity, structural diversity, and robust loading capacity, these novel materials have been hailed as star materials [52,53]. It is expected that novel MOFs that are more similar to natural enzymes in terms of their functions and structures can be developed in the future. Single-atom nanozymes, on the other hand, refer to nanomaterials in which the active sites consist of isolated single atoms, achieving atomic-level dispersion, maximizing atomic utilization, and resulting in superior catalytic activity [54]. Ji et al. [55] precisely engineered the active sites of iron-based single-atom nanozymes (e.g., FeN_4_, FeN_5_, or FeN_3_P configurations), enabling their catalytic efficiency to rival that of natural enzymes. In a separate study, Cheng et al. [56] reported a non-metal selenium single-atom nanozyme (SeSAE) as a safe and effective alternative for tumor therapy, demonstrating robust NADPH oxidase-like activity.

Carbon-based nanozymes are nanomaterials composed of non-metal elements or compounds [57]. These nanozymes do not rely on metal centers and, due to their unique physicochemical properties and biocompatibility, exhibit tremendous potential in mimicking enzyme activities. Moreover, carbon-based nanomaterials create microenvironments similar to the extracellular matrix of biological cells, indicating broad prospects for their application in studies of in vivo biodistribution and metabolic adaptability [58]. For instance, Fan et al. [59] developed a nanozyme with modulated ROS-like enzyme activity in nitrogen-doped porous carbon nanospheres to promote ROS production in lysosomes and thereby inhibit tumour proliferation in vivo. Currently known carbon-based nanomaterials with enzyme-like activities (CNMs) include fullerenes [60], carbon nanotubes [61], graphene [62], carbon dots [63], and other carbon-based nanomaterials. The unique enzymatic properties and advantages of carbon-based nanozymes continue to attract attention and are expected to remain a dynamic and challenging field in the coming years.

Innovative material design and development have led to groundbreaking advances in the field of nanozymes, significantly expanding their potential in applications such as biosensing, environmental remediation, and disease treatment. However, most nanozymes currently incorporate metal elements, and some metal-based nanozymes may exhibit potential toxicity. Their regulatory strategies and catalytic mechanisms still require further investigation, and enhancing their biosafety and biocompatibility remains a significant challenge.

**Table 1:** Nanozymes classified based on different core structures and representative examples.

Classification Based on Core Structure	Examples	Advantages	Disadvantages
Metal-based Nanozymes	Metal nanoparticles (e.g., gold, silver, copper) [40]	Excellent catalytic activity, good stability, and multifunctionality (such as conductivity and magnetism).	High metal costs, potential biological toxicity, and complex catalytic mechanisms.
Metal Oxide-based Nanozymes	TiO_2_, ZnO, CeO_2_ [43,44], Fe_3_O_4_, Fe_2_O_3_ [48]	Similar to metal nanozymes, but with higher biocompatibility.	Stability needs to be enhanced (such as aggregation, precipitation, or degradation).
Single-atom Nanozymes	Pt-N-C, Zn-N-C, Cu-N-C, Co-N-C, Fe-N-C [55], Se-N-C [56]	Low toxicity, high catalytic activity, distinct structure.	Hard to prepare and narrow substrate specificity.
Metal-Organic Framework-based Nanozymes	MOFs (e.g., ZIF-8 [51], UiO-66 [51]	Good biocompatibility, tunable properties, easy to functionalize, and multi-nanozyme mimicry.	Complex preparation, limited substrate specificity, and poor structural stability.
Carbon-based Nanozymes	Fullerene [60], Carbon Nanotubes (CNTs) [61], Graphene [62], Carbon Quantum Dots (CQDs) [64]	Good biocompatibility and degradability, abundant raw material sources, low cost, tunable structure, and surface chemical properties.	The catalytic activity still lags behind that of natural enzymes.
Covalent organic framework, COF	l-His100@Fe-COF [63], CoP-TPE-COF, COF@Co_3_O_4_	High specific surface area and porosity, easy to functionalize, structural designability, biocompatibility, and degradability.	Complex preparation process, cost and scalability issues, and stability problems.

### 2.2. The Preparation of Nanozymes and Associated Challenges

Currently, nanozymes are primarily synthesized through methods such as co-precipitation, hydrothermal/solvothermal processes, sol-gel techniques, and template-assisted strategies [65]. While these approaches offer flexibility in tailoring size, morphology, and composition, they often suffer from issues related to reproducibility and scalability. For instance, hydrothermal methods may yield products with batch-to-batch variability due to subtle changes in reaction parameters, making standardization difficult for large-scale production.

Another significant limitation is the stability of nanozymes under physiological or industrial conditions. Surface oxidation, aggregation, or leaching of active components can compromise long-term activity and biocompatibility. Moreover, the cost of precursors and the need for specialized equipment, particularly in template-based or multi-step synthetic routes, can elevate production expenses and hinder commercialization [66]. In addition, controlling the uniform distribution of active sites and achieving consistent catalytic performance across different batches remains a formidable task.

Addressing these challenges requires the development of more robust, reproducible, and environmentally benign synthesis protocols, ideally through green chemistry approaches [67]. Further, integrating real-time monitoring and quality control during fabrication could enhance reproducibility and scalability. A comprehensive understanding of these synthetic limitations is essential not only for optimizing nanozyme performance, but also for facilitating their translation from the laboratory to practical applications in diagnostics, therapeutics, and environmental remediation.

### 2.3. Regulation of Nanozymes Activity

Despite the advantages of nanozymes, which are more stable and economical than natural enzymes, the catalytic efficiency of many nanozymes is still lower than that of natural enzymes. To address this issue, the tunability of nanozymes can be exploited for rational optimization, enabling the construction of nanozymes with enhanced catalytic activity that may even rival that of natural enzymes. For example, altering particle size, incorporating heteroatom doping, and mimicking the active centers of natural enzymes are effective strategies for modulating the catalytic performance of nanozymes.

**Figure 2:**
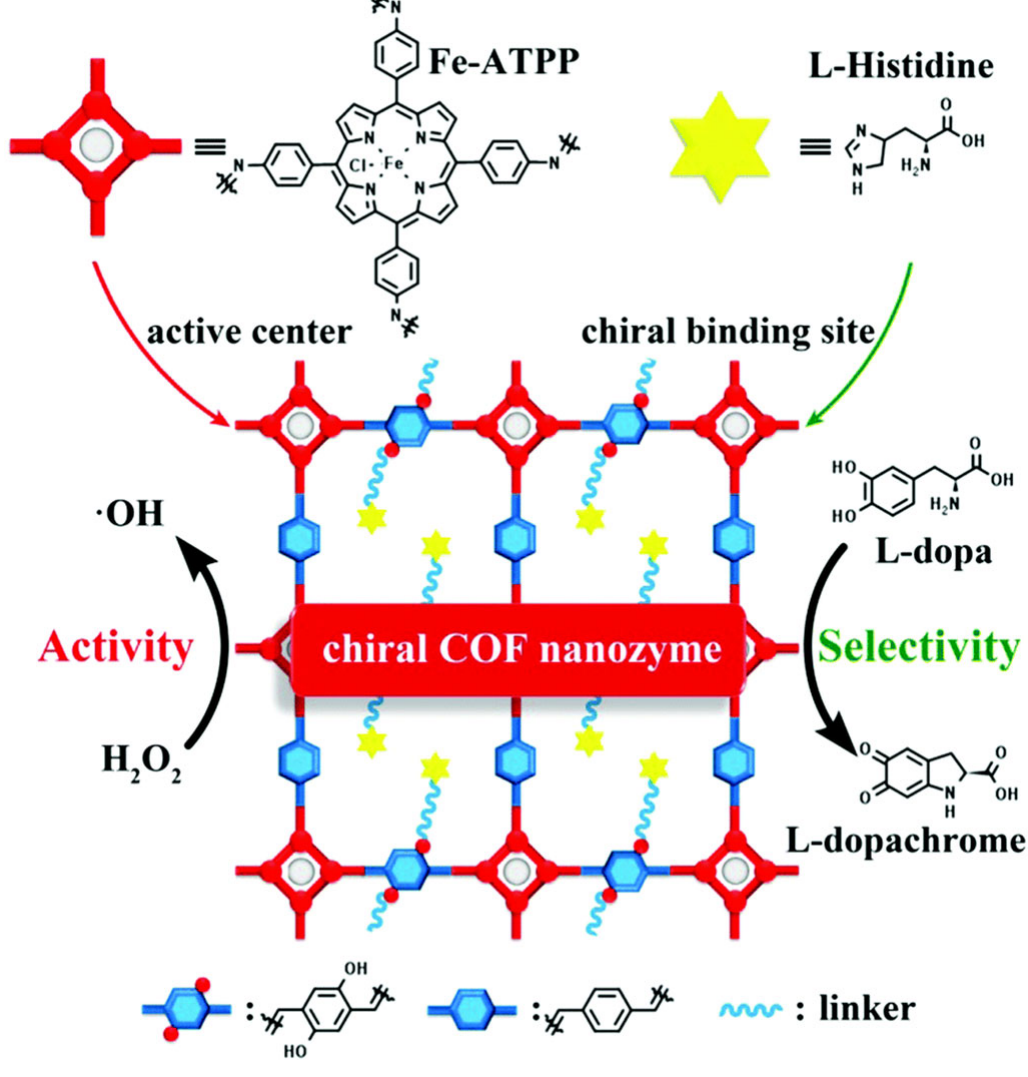
Chiral COF nanozymes functionalized with l-histidine (l-His) [63].

Reducing the size of nanozymes increases their specific surface area, thereby exposing more active sites for substrate interaction and ultimately enhancing catalytic efficiency [68]. For example, under identical conditions, 30 nm iron oxide nanoparticles (IONPs) exhibit significantly stronger peroxidase (POD) activity compared to their 150 nm and 300 nm counterparts. Similarly, 43 nm Prussian blue nanozymes (PBNZs) demonstrate higher POD- and catalase (CAT)-like activities than 81 nm PBNZs, owing to their larger specific surface area [69]. Furthermore, heteroatom doping provides a precise means to modulate both the activity and selectivity of nanozymes, representing a promising strategy with broad application prospects. Studies have revealed that while certain carbon-based nanozymes exhibit relatively weak enzyme-mimicking activities and limited intrinsic catalytic potential, their performance is largely governed by surface functional groups and unique electronic structures. Thus, heteroatom doping of carbon nanomaterials has emerged as an effective approach to developing high-performance carbon-based nanozymes [70]. Tao et al. [71] constructed Cu, N co-doped hollow carbon nanospheres (CuNHCNs) that feature highly active Cu–Nx sites, endowing them with high affinities for 3,3′,5,5′-tetramethylbenzidine (TMB) and H_2_O_2_, with Km values of 0.0655 mM and 0.918 mM, respectively. In detection assays, these CuNHCNs exhibited exceptional POD simulation activity and sensitivity, opening a novel, cost-effective pathway for the synthesis of high-performance nanozymes. The superior catalytic efficiency of natural enzymes is largely attributed to the spatial architecture of their active centers, where unique substrate-binding pockets and the modification of amino acid residues play critical roles. Inspired by this, researchers have designed a series of biomimetic nanozymes by emulating the structural features and catalytic mechanisms of natural enzyme active centers. In 2017, Fan Kelong and his team [72] mimicked the structure of histidine residues (His42 and His170) in the active centre of horseradish peroxidase (HRP) and covalently modified histidine residues on the surface of Fe_3_O_4_ to form the His-Fe_3_O_4_ nanoenzyme. The modified His-Fe_3_O_4_ had a higher affinity for H_2_O_2_ and increased the catalytic efficiency nearly 20-fold, which greatly enhanced the POD activity. In 2019, inspired by the iron porphyrin structure at the active center of natural peroxidases, the team used the metal–organic framework (MOF) material ZIF-8 as a precursor to design and synthesize a high-activity single-atom carbon nanozyme (PMCS) featuring a Zn porphyrin structure. PMCS SAzyme demonstrated excellent biocompatibility and achieved an inhibition rate of up to 99.87% against Pseudomonas aeruginosa, once again opening new horizons in the design of biomimetic enzymes and the field of biocatalysis [73]. Through persistent efforts by researchers, the catalytic activity of certain modified nanozymes has, in some cases, matched or even surpassed that of natural enzymes. Zhou et al. [63] reported the first chiral nanozyme exhibiting higher activity than natural enzymes. Covalent organic frameworks (COFs), a novel class of nanomaterials characterized by high specific surface area and porosity, were endowed with enhanced activity and selectivity through the incorporation of iron porphyrin and L-histidine (L-His) units. Moreover, by varying the L-His content, it is possible to finely tune the activity and selectivity of the COF nanozyme as well as control the catalytic reaction process. The modified COF nanozyme achieved POD activity up to 21.7 times that of its natural counterpart (Figure 2).

## 3. Application of Nanozymes in POCT

The detection mechanism of POCT is to specifically identify the target analytes and convert them into detectable output signals through various physicochemical reactions [74]. Currently, enzymes are the most widely used components and are indispensable in POCT detection technologies [75]. The catalytic mechanism and surface structure of enzymes play a decisive role in determining both detection performance and the scope of their applications [76]. Nanozymes with diverse enzyme-mimicking activities can catalyze substrates to generate a range of signals—including light, heat, color, electrical, or pressure signals—which enables a variety of detection strategies such as chemiluminescence, electrochemical, colorimetric, fluorescence, Raman scattering, and pressure sensing methods [77]. POCT devices can capture, process, and directly display these signals for target detection. Notably, by leveraging the size effects of nanomaterials to amplify signals, these devices have significantly enhanced sensor sensitivity, detection efficiency, and overall performance [21]. As an innovative technology, nanozymes have broadened the application fields of POCT and spurred technological innovation in biosensing. In this review, we systematically elucidate the principles underlying various detection techniques—including colorimetric analysis, chemiluminescence, fluorescence, electrochemical methods, and SERS—and discuss the application potential of different nanozyme-based POCT detection strategies along with prospects for their future development (Table 2).

### 3.1. Colorimetric Sensing Based on Nanozymes

Colorimetric detection refers to the qualitative or quantitative measurement of analytes based on the color change produced by a chemical reaction, with the intensity of the color change being directly proportional to the concentration of the target analyte [78]. As one of the most significant sensing technologies, colorimetric POCT offers numerous advantages including high selectivity, robust practicality, ease of observation, low cost, imaging capability, and compatibility with portable devices. Consequently, it eliminates the need for complex instrumentation and expensive equipment, rendering it highly suitable for on-site analysis and POCT applications [79,80]. At present, it has been widely applied in various fields, including environmental monitoring, forensic evidence analysis, disease diagnosis, food testing, and biomarker detection [81,82,83,84]. The reaction between nanozymes and substrates can induce the generation and alteration of color, primarily by leveraging their peroxidase- or oxidase-like activities. Common substrates are typically redox-active molecules, such as 3,3′,5,5′-tetramethylbenzidine (TMB), 2,2′-azino-bis(3-ethylbenzothiazoline-6-sulfonic acid) (ABTS), and o-phenylenediamine (OPD), which serve as chromogenic probes to produce colorimetric output signals [85]. These signals, manifested as distinct colors, enable qualitative or semi-quantitative analysis via direct visual inspection; for example, the oxidation product of TMB is blue, that of ABTS is green, and that of OPD is yellow [86].

**Figure 3:**
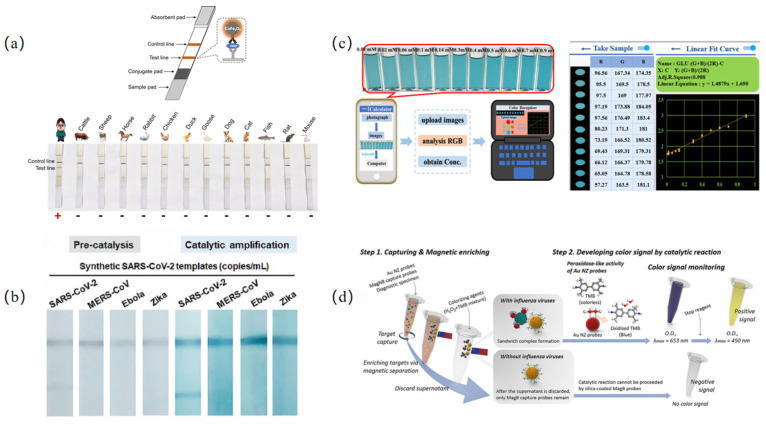
Colorimetric POCT platforms based on nanozymes. (**a**) Schematic illustration for CoFe_2_O_4_ nanozyme strip [87]. (**b**) FeS_2_ nanozyme strip for nucleic acid detection of SARS-CoV-2. FeS_2_ nanozyme strip for nucleic acid detection of other RNA viruses [88]. (**c**) Colorimetric analysis of different glucose concentrations using Fe@NCMzyme POCT platform [89]. (**d**) Working principle for the quantification of influenza viruses using MagLISA-based colorimetric diagnostics kit [91].

Bloodstains are among the most critical pieces of physical evidence at crime scenes, playing a vital role in evidence collection, case analysis, and forensic identification. Hong et al. [87] doped cobalt (Co) into Fe_3_O_4_ nanoparticles to prepare CoFe_2_O_4_ nanozymes with high peroxidase-like catalytic activity. The nanozymes were further modified with carboxyl groups, and subsequently developed into CoFe_2_O_4_ nanozyme test strips for the detection of human hemoglobin (HGB). The detection limit reached as low as 1 ng/mL, significantly improving sensitivity and versatility compared to conventional colloidal gold strips. In addition, the nanozyme strips can detect artificially obliterated bloodstains, which cannot be met by colloidal gold strips. This test strip, as an effective on-site detection method for human bloodstains, has made a significant contribution to criminal case investigations (Figure 3a). Meng et al. [88] reported a nanozyme-based test strip that integrates recombinase polymerase amplification (RPA) with FeS_2_ for the detection of SARS-CoV-2. This approach leverages the rapid amplification and low reaction temperature (37–42 °C) of RPA, along with the POD-like activity of FeS_2_, to catalyze TMB substrate oxidation and enhance the colorimetric signal on the test strip. The method achieved a nucleic acid detection limit of 200 copies/mL for SARS-CoV-2, with the entire process completed within 36 min, significantly reducing detection time while improving sensitivity (Figure 3b). Liu et al. [89] developed an Fe@NCMzyme-based colorimetric sensing platform with peroxidase-like activity for on-site glucose detection in food samples. In this three-dimensional (3D)-structured nanozyme, Fe_3_O_4_ nanoparticles (FeNPs) serve as the catalytic center, while nitrogen-doped carbon nanofiber microspheres (NCMs) function as binding sites. The 3D porous architecture of Fe@NCMzyme facilitates electron transfer, enhancing nanozyme activity and compensating for the lower catalytic efficiency of nanozymes compared to natural enzymes. Furthermore, when integrated with a laptop-based POCT platform, this system achieved a glucose detection limit of 3.125 μM, demonstrating its significant potential for glucose detection in both food safety and medical diagnostics (Figure 3c). Influenza is an acute respiratory infection caused by the influenza virus and is one of the most common infectious diseases of the respiratory tract [90]. Therefore, developing a rapid, sensitive, and accurate POCT platform for influenza viruses is of great importance in reducing viral transmission and facilitating clinical treatment. Lee et al. [91] developed a nanozyme-linked immunosorbent assay (MagLISA) based on magnetic MagNBs, providing a simple, sensitive, and precise colorimetric POCT method for influenza virus detection. This approach utilizes gold nanozymes (AuNZs) as probes, leveraging their POD-like activity to generate a colorimetric signal for the highly sensitive detection of the H1N1 influenza virus. The sensitivity of this sensing platform is 2000 times higher than that of commercial test kits and 400 times higher than that of conventional ELISA assays (Figure 3d).

### 3.2. Electrochemical Sensing Based on Nanozymes

Electrochemical sensing, with its ability to output signals based on electrochemical responses, offers significant advantages in various aspects, such as intelligence, miniaturization, easy integration, high sensitivity, rapid accuracy, and low cost [92]. Electrochemical biosensors generate currents during the measurement process, and these currents are positively correlated with the concentration of the analyte or its production/consumption rate. By measuring the changes in current or voltage at the electrode surface, quantitative analysis of the analyte can be performed [93]. The efficiency of the biomolecular recognition element is crucial to the effectiveness and signal sensing performance of electrochemical biosensors [94].

The powerful physicochemical properties and catalytic capabilities of nanozymes can enhance the electrochemical signals generated during sensing, reduce the detection threshold of the sensor, increase sensitivity, and provide a shorter response time along with good real-time monitoring capabilities. These attributes make nanozymes particularly suitable for the design of small, portable, and rapid on-site detection devices, offering significant potential in the development of POCT [95]. For instance, Ko et al. [96] developed an electrochemical microfluidic POCT device based on a Au@PtNP/GO nanozyme by synthesizing a hybrid nanostructure of graphene oxide (GO) and bimetallic nanoparticles (Au and Pt). The POD activity of the Au@PtNP/GO nanozyme can oxidize TMB, and the current generated by the oxidation of TMB shows a linear relationship with the concentration of H_2_O_2_. Compared to colorimetric detection methods, it offers a wider detection range and a lower detectable concentration of H_2_O_2_, with a limit of detection (LOD) of 1.62 μM. The developed POCT device can accurately detect the H_2_O_2_ content in samples and exhibits good reproducibility.

POCT applications are often targeted at grassroots healthcare and at-home self-testing scenarios. Currently, single electrochemical POCT sensing platforms based on nanozymes are relatively rare, primarily because the electrochemical signal-based detection results often lack intuitive and easily interpretable characteristics, which limits their widespread adoption and practicality. Researchers typically combine electrochemical sensing with colorimetric sensing to form electrochemical–colorimetric dual-mode sensing platforms. This combination integrates the quantitative advantages of electrochemical sensing with the intuitive nature of colorimetric sensing, offering higher sensitivity, accuracy, and multi-detection capabilities. It compensates for the limitations of single electrochemical sensors and makes such platforms more widely applicable in POCT.

### 3.3. Fluorescent Analysis and Detection Based on Nanozymes

There are various methods for fluorescence analysis and detection based on nanozymes. One approach involves combining nanozymes with fluorophores to construct dual-functional nanomaterials that possess both catalytic activity and the ability to emit fluorescent signals [97]. Another method utilizes nanozymes to directly catalyze fluorescent substrates, producing fluorescent signals for the qualitative and quantitative analysis of target molecules [98]. Additionally, some approaches involve the catalytic reactions of nanozymes to generate intermediate products that indirectly produce fluorescent probes. For example, nanozymes with POD-like activity can catalyze H_2_O_2_ to generate hydroxyl radicals (·OH), which can then react with precursor molecules of fluorescent probes, resulting in the formation of fluorescent probes with characteristic fluorescence properties [99]. In fluorescence sensing strategies, fluorophores typically respond to target analytes through fluorescence quenching or enhancement, a phenomenon that is critical in fluorescence sensing. However, this response is easily influenced by excitation intensity and external environmental factors. Ratio fluorescence involves the incorporation of nanoparticles or organic dyes into composite materials, as well as the synthesis of dual-emission fluorophores, which provide built-in calibration functions. This approach enhances the accuracy and reliability of measurements and has gained significant interest from researchers in the fields of biomarker detection, imaging, and sensing [100].

**Figure 4:**
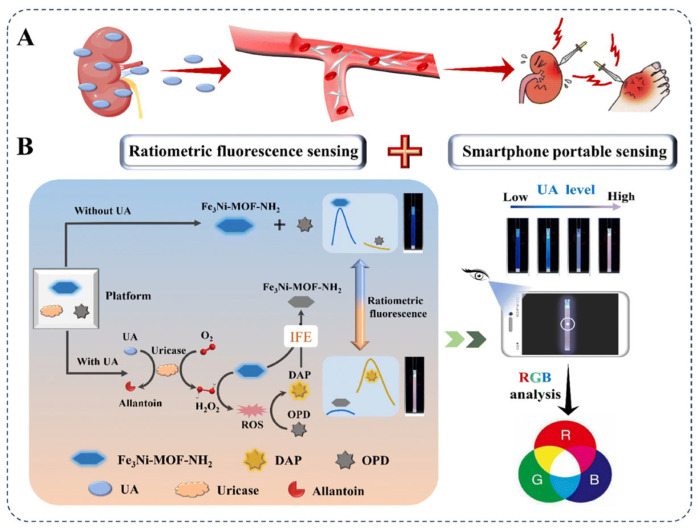
(**A**) Schematic illustration of the accumulation process of UA in the human body. (**B**) Mechanism of the ratiometric fluorescent biosensor for UA based on the Fe_3_Ni-MOF-NH_2_ nanozyme and its smartphone-based portable sensing application [101].

Han et al. [101] reported the first ratiometric fluorescence sensor for uric acid (UA) based on Fe_3_Ni-MOF-NH_2_ as an MOF nanozyme mimic and developed a smartphone-assisted portable sensing platform. This platform exploits the peroxidase-like activity and inherent fluorescence properties of Fe_3_Ni-MOF-NH_2_. In the absence of UA, the MOF displays fluorescence only at 430 nm (FI_430_); however, upon the addition of UA, a UA/uricase-catalyzed reaction is initiated, generating H_2_O_2_. Under the catalytic influence of the MOF nanozyme, the produced H_2_O_2_ oxidizes o-phenylenediamine to yield highly fluorescent 2,3-diaminophenazine (DAP), which emits strong fluorescence at 565 nm (FI_565_), resulting in a low FI_430_ signal and a high FI_565_ signal. The cascade catalytic reaction of UA/uricase/o-phenylenediamine, when integrated with POCT, ultimately achieves a detection limit of 24 nM for UA, thereby laying the foundation for the development of multifunctional fluorescence biosensors based on nanozymes and enabling portable, efficient, and real-time UA detection (Figure 4).

A paper-based platform has been employed to immobilize fluorophores for POCT analysis, offering a cost-effective and user-friendly detection method. Wang et al. [102] developed a portable, fluorescence-based paper hydrogel sensor for the precise monitoring of captopril. The paper hydrogel platform comprises UiO-66-NH_2_@ZIF-8 and Co, N-doped nanozyme materials. When the Co, N-doped nanozyme catalyzes the oxidation of the chromogenic substrate DPD, the resulting oxidized DPD produces a visually detectable pink signal that in turn quenches the fluorescence of UiO-66-NH_2_@ZIF-8. In the presence of captopril, however, the oxidation of DPD by the Co, N-doped nanozyme in the hydrogel-assisted paper sensor is inhibited, leading to a change in color and the recovery of the fluorescence intensity of UiO-66-NH_2_@ZIF-8. By employing smartphone photography and subsequent analysis with ImageJ software, the image parameters are converted into quantitative data, thereby enabling the quantitative detection of captopril.

**Figure 5:**
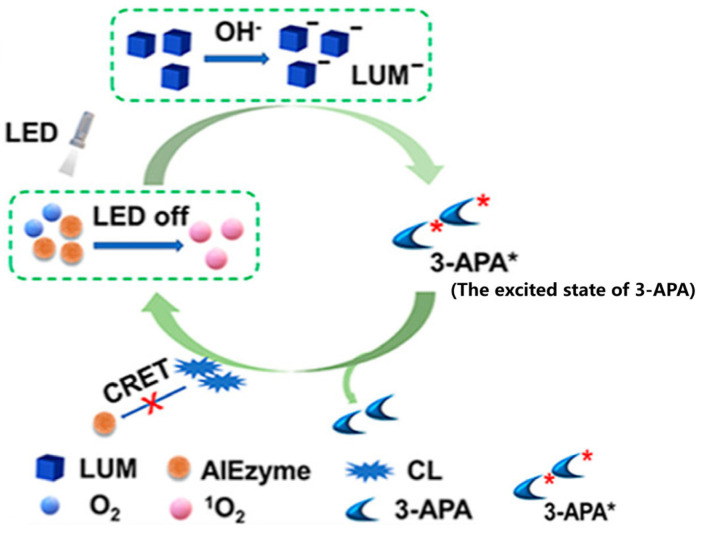
Possible mechanism proposed for long-lasting CL of AIEzyme/LUM [103].

Wu et al. [103] constructed an innovative POCT platform for highly sensitive bacterial detection by leveraging a chemiluminescence-driven ratiometric fluorescence sensor. This system employs an aggregation-induced emission molecule, FI430, endowed with intrinsic oxidase-like activity (termed “AIEzyme”). Furthermore, a fluorescent probe (FL) is encapsulated within ZIF-8 to form a ZIF@FL composite, which is then seamlessly integrated with the AIEzyme/LUM system to establish the complete sensing platform. In this system, the self-cycling reactive oxygen species (ROS) generated by AIEzyme enables luminol to continuously produce chemiluminescence (CL) for approximately 120 min even in the absence of H_2_O_2_. This sustained CL, in turn, excites FL, allowing its emission to persist for around 50 min without any external light source, thereby forming an afterglow luminescence system that exhibits enhanced anti-interference capability compared to traditional fluorescence emission methods. Upon incubation with lysozyme, the target *Escherichia coli* undergoes lysis, releasing ATP which subsequently triggers the decomposition of ZIF-8 and the release of the encapsulated fluorescent probe. Quantitative detection of *E. coli* is then achieved via ratiometric fluorescence analysis using a smartphone-based readout. The prolonged luminescence and ratiometric strategy significantly enhance detection accuracy, and the H_2_O_2_-free, self-sustaining system—requiring no external excitation—holds considerable potential for clinical diagnostic applications (Figure 5).

### 3.4. Chemiluminescence Detection Based on Nanozymes

In nanozyme-based chemiluminescence detection, the nanozyme serves as a catalyst that facilitates the reaction of a chemiluminescent substrate (e.g., luminol) to emit light in the presence of an activator (e.g., hydrogen peroxide, H_2_O_2_). The intensity of the emitted light is directly proportional to the concentration of the analyte, thereby enabling quantitative analysis through the conversion of light signals into analyte concentration [104]. Unlike fluorescent analytical assays, CL assays do not require excitation from a light source, have low background interference, and provide a continuous light signal, which is currently very promising for the detection of pharmaceuticals as well as various bioenergetic markers.

**Figure 6:**
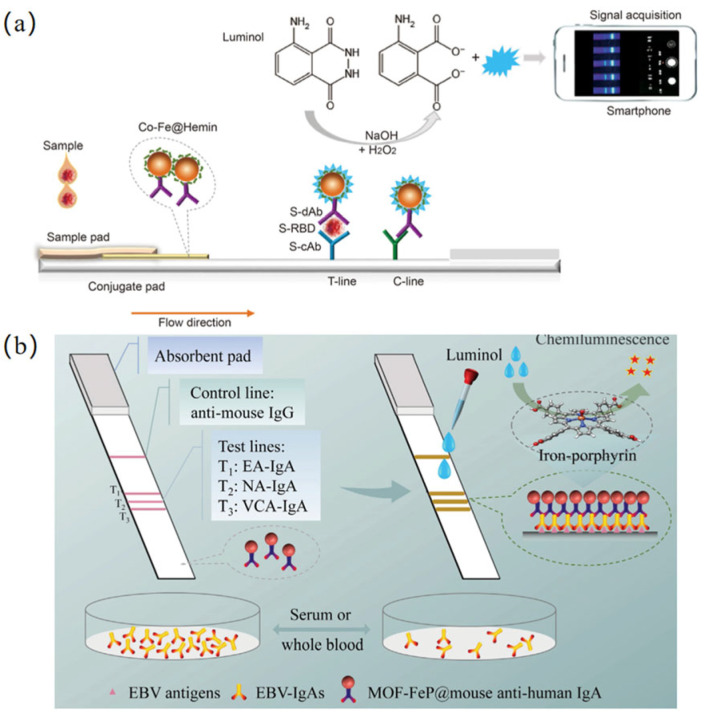
Nanozyme-based chemiluminescence detection platform. (**a**) Schematic illustration of the nanozyme chemiluminescence paper test for SARS-CoV-2 S-RBD antigen. Recognition, separation, and catalytic amplification by nanozyme probes [105]. (**b**) Schematic illustration of the preparation of MOF-FeP based strip and its application in detection of EBV-lgAs [106].

The novel coronavirus disease caused by severe acute respiratory syndrome coronavirus 2 (SARS-CoV-2) has recently escalated into a global pandemic, posing a severe threat to public health worldwide. Liu et al. [105] developed a chemiluminescent test strip based on a Co-Fe@hemin peroxidase nanozyme for the rapid and highly efficient detection of the SARS-CoV-2 spike antigen. This test strip exhibits exceptionally high specificity, with no cross-reactivity observed with other coronaviruses or influenza A subtypes. Moreover, the entire assay can be completed within 16 min, featuring a detection limit of 0.1 ng/mL for the recombinant spike antigen and a linear range of 0.2–100 ng/mL. This approach seamlessly integrates nanozyme catalysis, lateral flow immunoassay, and chemiluminescence sensing to establish a novel POCT platform (Figure 6a).

Nasopharyngeal carcinoma (NPC) is a malignant tumor that is difficult to diagnose at an early stage and is associated with high mortality in advanced stages. Epstein–Barr virus (EBV) has been confirmed as a reliable biomarker for an increased risk of NPC. Wang et al. [106] synthesized a single-atom nanozyme (SANzyme) based on a ferriporphyrin-based metal–organic framework (MOF-FeP) for the detection of EBV-IgA. By harnessing the catalytic chemiluminescence of MOF-FeP, the research team developed a chemiluminescent test strip capable of simultaneously detecting three types of EBV-IgA antibodies. The assay can be completed within 16 min, is applicable to whole blood samples, and remains unaffected by potential interfering impurities, thereby providing a robust platform for the early screening and diagnosis of EBV-associated NPC (Figure 6b).

### 3.5. SERS Sensor Based on Nanozymes

Raman spectroscopy is a non-destructive detection technique that employs photons as the probing tool and is based on the phenomenon of Raman scattering. It provides unique “fingerprint” information on chemical and biomolecular structures, thereby enabling molecular identification and real-time detection, and has become an essential tool in analytical chemistry [107,108]. However, due to the inherently low cross-section of Raman scattering, its signal is intrinsically very weak, which limits its broader application [109]. It was not until 1974 that Fleischmann et al. discovered SERS [110]; SERS can significantly amplify Raman signals, achieving enhancements of 10^4^–10^10^ times when analytes are in close proximity to or adsorbed onto metallic nanomaterials such as Au, Ag, or Cu. The SERS technique offers high sensitivity, convenience of use, multidimensional analysis, low sample consumption, and the capability for in situ detection [111], making it widely applicable across various fields [112,113].

**Figure 7:**
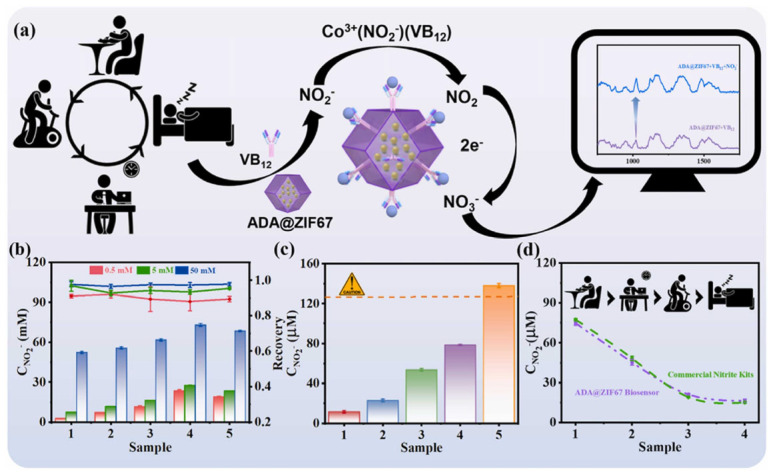
(**a**) ADA@ZIF67 diagram of nitrite detection when biosensors are used in human activities. (**b**) Detection values and recovery rates of nitrite with different concentrations (0.5 mM, 5 mM, 50 mM) added to food samples. (**c**) Determination of nitrite content in saliva samples of volunteers after eating different samples. The yellow bar indicates the threshold for nitrite concentration. (**d**) ADA@ZIF67 biosensor and commercial kit compared the volunteers’ nitrite levels during different activities [114].

In SERS sensing, both surface chemical behavior and surface plasmon resonance (SPR) performance is crucial. Metal–organic frameworks (MOFs), as porous solid nanozymes with advantages in microstructure, stability, and photocatalytic performance, can facilitate the aggregation of noble metal nanoparticles (NPs). NPs derived from MOFs can enhance the localized surface plasmon resonance (LSPR) effect and thereby amplify the SERS signal. Zeng et al. [114] developed a metal–organic framework (ZIF-67)-based nanozyme incorporating the biomimetic enzyme cyanocobalamin (VB12), which was subsequently employed as an ion-selective SERS instant detection platform (ADA@ZIF-67 biomimetic enzyme biosensor) for the rapid and sensitive detection of nitrite. In this system, the VB12 biomimetic enzyme forms an ion-selective layer that specifically captures and oxidizes nitrite. The cobalt-based MOF-derived nanoparticles serve to immobilize the biomimetic enzyme and promote electron transfer generated by redox reactions on the ion-selective layer, thereby achieving high sensitivity and selectivity. When utilized as a POCT reagent, the SERS sensor exhibited a nitrite detection limit of 1.67 nM, demonstrating that nanozyme-based SERS sensors offer substantial advantages in the POCT field and hold great promise as potential tools for detection and healthcare applications (Figure 7).

### 3.6. Other Detection Methods and Sensing Strategies Based on Nanozymes

#### 3.6.1. Pressure Sensor

**Figure 8:**
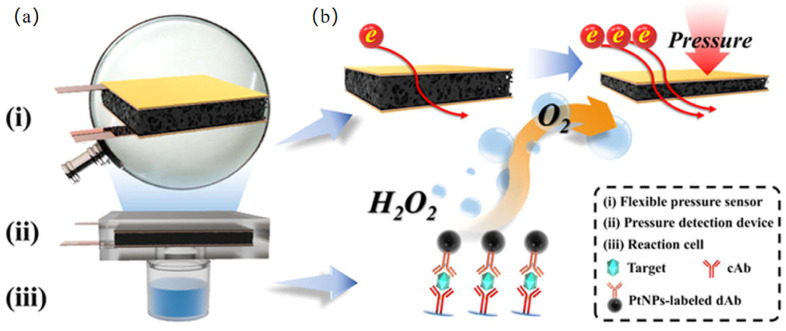
Schematic Illustration of the (**a**) pressure-based immunosensing device and (**b**) working principle of the pressure-based immunoassay for CEA [118].

In addition to the detection methods mentioned above, other promising nanozyme-based sensing strategies are under development. In recent years, with rapid advancements in technology, wearable pressure sensors have demonstrated significant potential for smart, real-time detection and human–machine interaction [115]. Typically, the decomposition of hydrogen peroxide (H_2_O_2_) produces oxygen (O_2_) and water (H_2_O). In a confined environment, the accumulation of O_2_ leads to an increase in pressure; owing to its high yield and non-toxic nature, O_2_ is ideal for POCT pressure sensors, where a portable handheld pressure meter can readily detect the signal. It is well established that the catalase-like (CAT) activity of nanozymes can catalyze the generation of O_2_ from H_2_O_2_. In particular, platinum (Pt) and other platinum-group metals, due to their excellent catalytic properties, can significantly amplify the sensing signal in gas pressure detection, making them highly promising peroxidase-like nanozymes for H_2_O_2_-related applications [116,117]. The integration of pressure-sensing technology into POCT devices offers considerable advantages. Yu et al. [118] exploited the unique properties of platinum nanoparticles (PtNPs) to develop a flexible pressure sensor featuring a three-dimensional polypyrrole (3DPPy) foam as the sensing layer for target analyte concentration detection. In a sealed system, PtNPs catalyze the decomposition of hydrogen peroxide (H_2_O_2_) into oxygen (O_2_); as the generated gas increases the internal pressure, the 3DPPy foam is compressed, leading to a decrease in resistance. By monitoring the corresponding electrical signal changes, the flexible pressure sensor constructed from 3DPPy foam can rapidly and sensitively detect the concentration of carcinoembryonic antigen (CEA), with a detection limit of 0.13 ng/mL. The utilized 3DPPy foam board demonstrates excellent mechanical flexibility, compressibility, conductivity, and elasticity, enabling precise and rapid monitoring of pressure signals (Figure 8). The high sensitivity, flexibility, stability, and durability of this flexible pressure sensor hold significant implications for pressure-based POCT biosensing. However, current reports on gas-pressure biosensors are limited to sensors exhibiting specific interactions, and there remains a need for further research in analyzing complex samples, as well as addressing challenges related to the limited gas-generation reaction and the safety and stability of the materials used [119].

#### 3.6.2. Multi-Mode Platform

Compared with single technologies, the integration or combination of two or more analytical techniques offers significant advantages. Dual-mode platforms can overcome the limitations inherent in single-mode approaches through their complementary nature, thereby enhancing the sensor’s adaptability to complex samples, particularly in challenging matrices such as blood and urine. In response to various detection requirements, flexible integration of multiple modalities—such as electrochemical, colorimetric, fluorescence, chemiluminescence (CL), and photothermal (PT) methods—has proven highly effective in POCT applications. To date, numerous dual-mode sensing platforms based on two different or multiple relatively independent signal transduction mechanisms have been developed.

**Figure 9:**
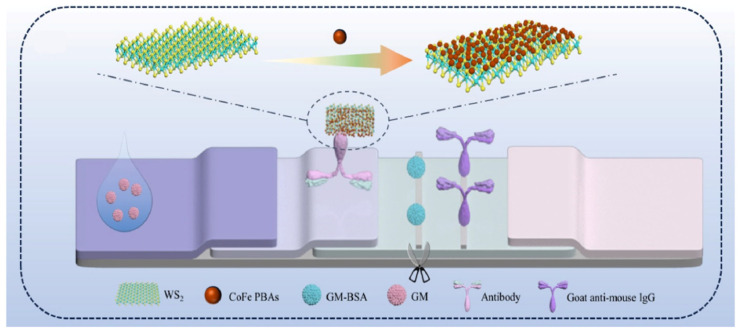
Construction of dual-signal mode LFIA for GM [120].

Wu et al. [120] developed a dual-mode lateral flow immunoassay (LFIA) for the detection of gentamicin (GM), which is based on a CoFe PBA/WS_2_ nanozyme-mediated chemiluminescence (CL) and photothermal (PT) approach. By evaluating the scavenging capabilities of ascorbic acid, thiourea, hydroquinone, and tryptophan for reactive oxygen species (ROS), it was confirmed that the CoFe PBA/WS_2_ nanozyme, possessing peroxidase-like activity, can catalyze the luminol–H_2_O_2_ system to generate CL and significantly enhance the luminol CL reaction by approximately 2026-fold. Simultaneously, this nanozyme effectively mediates an enhanced PT effect. The complementary features of the CL-LFIA and PT-LFIA provide the POCT sensor with robust anti-interference capabilities, achieving detection limits for GM of 0.33 and 16.67 pg/mL, respectively. This dual-mode POCT sensor exhibits broad potential for applications in drug concentration monitoring and clinical diagnostics (Figure 9).

**Figure 10:**
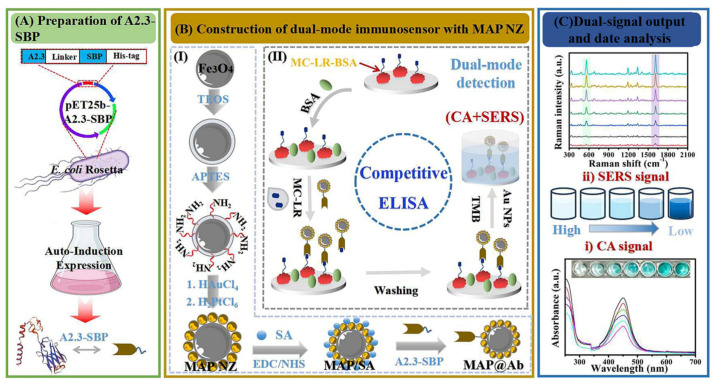
Schematic illustration of the colorimetric-SERS dual-mode immunosensor for rapid detection of MC-LR based on MAP@Ab probes catalyzed by TMB (CA signal for the colorimetric immunoassay and Au NPs enhanced signal for SERS detection) [121].

In colorimetric sensing, the signal generated by the oxidized substrate TMB (oxTMB) can be collected using Raman spectroscopy and gold nanoparticles (Au NPs) to enhance the signal, thereby further improving the sensitivity of SERS. Wu et al. [121] designed a dual-functional nanobody (A2.3-SBP) composed of a nanobody and a streptavidin-binding peptide, and integrated it with Fe_3_O_4_@Au-Pt nanozymes to develop an on-site detection platform that incorporates both colorimetric and SERS sensing for microcystin-LR (MC-LR). This platform achieved dual-signal detection of MC-LR in 96 water samples (0.03 μg/kg) within 30 min, with detection limits of 0.26 and 0.032 ng/mL, respectively. The method’s specificity and robustness make it an effective POCT tool for environmental monitoring, holding great promise for significant contributions to environmental protection and water resource utilization (Figure 10).

**Figure 11:**
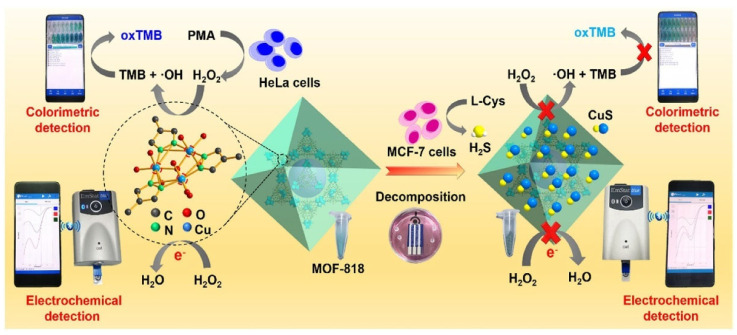
MOF-818 nanozyme-based colorimetric and electrochemical dual-mode smartphone sensing platform for in situ detection of H_2_O_2_ and H_2_S released from living cells [122].

A smartphone-based colorimetric-electrochemical sensing platform has been applied in POCT due to its simplicity, low cost, broad detection range, and high sensitivity. Yu et al. [122] developed a portable dual-mode sensor integrating both colorimetric and electrochemical detection. The sensor employs an MOF-818 nanozyme with excellent POD activity to detect hydrogen peroxide (H_2_O_2_) and hydrogen sulfide (H_2_S) in biological samples. Specifically, the hue and saturation values (HSVs) of the blue oxTMB solution produced via nanozyme-catalyzed POD activity were captured using a “Color Identifier” application on a smartphone. Simultaneously, the MOF-818 nanozyme exhibits remarkable electrocatalytic activity by reducing H_2_O_2_ to generate an electrical current. This platform facilitates in situ detection of H_2_O_2_ and H_2_S released from living cells, offering a novel method for cell monitoring and promising real-time clinical diagnostics (Figure 11).

Microfluidic devices offer several advantages, including low sample and reagent consumption, rapid detection, high integration, automated operation, and portability. These features enable highly sensitive and multifunctional rapid testing, making them particularly well-suited for POCT and applications in resource-limited settings. Yin et al. [123] developed a miniaturized and automated microfluidic device capable of dual-mode colorimetric and fluorescent detection of *Escherichia coli* (*E. coli*) in real samples, based on a Cu^2+^–o-phenylenediamine (OPD) reaction system. A smartphone was employed to capture the RGB data from the chip’s reaction chamber, enabling quantitative analysis of both colorimetric and fluorescence signals within a concentration range of 10^2^–10^6^ CFU mL^−1^. In addition, the use of an automated, portable, and compact microfluidic device overcomes the limitations of conventional detection methods, such as low efficiency and complex procedures. This system enables high-precision, fully automated detection and is well-suited for on-site POCT, offering an ideal solution for expanding the practical applications of *E. coli* detection.

Neurodegenerative diseases are a class of chronic disorders marked by the progressive damage and loss of neuronal function, with Alzheimer’s disease and Parkinson’s disease being prominent examples. Acetylcholinesterase (AChE) plays a vital role in maintaining neural homeostasis and is essential for regulating key physiological processes such as memory, learning, and muscle control. Dysregulation of AChE activity is closely linked to the development of various neurodegenerative conditions. Chen et al. [124] developed a highly sensitive and reliable dual-mode colorimetric/photothermal sensing platform for acetylcholinesterase (AChE) detection, based on iodide ion-synergized covalent porphyrin–triazine framework nanozymes (Zn-CTF/I). The introduction of iodide ions synergistically interacted with zinc centers, effectively modulating the electronic structure of the catalytic active sites. This interaction markedly enhanced the peroxidase-like (POD-like) activity of the nanozymes. As a result, the detection limit for AChE was reduced by an order of magnitude compared to control systems, achieving a minimum detection limit of 0.003 U L^−1^. This performance surpasses that of previously reported assays, demonstrating the significant potential of Zn-CTF/I nanozymes in ultrasensitive AChE detection. This dual-mode platform offers a powerful point-of-care diagnostic tool for the early detection and intervention of neurological disorders.

**Figure 12:**
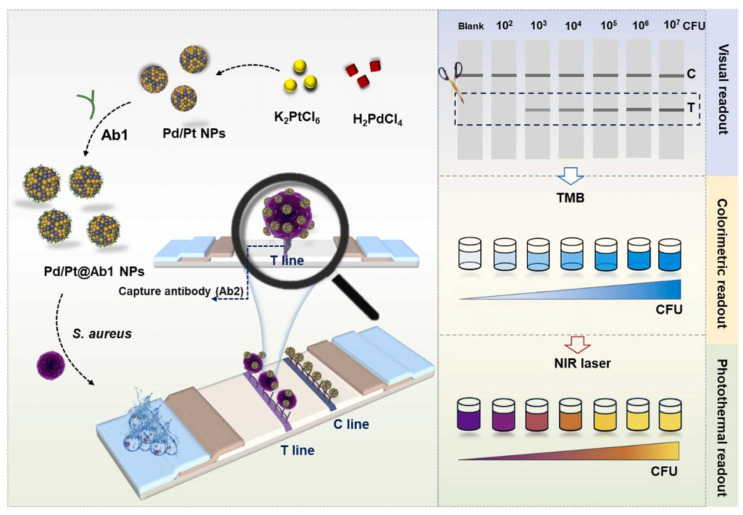
Schematic synthesis of Pd/Pt@Ab1 NPs and their application in sandwich paper-based multimodal LFIA detection of *S. aureus* with visual, colorimetric, and photothermal signal outputs [125].

Liu et al. and his team [125] synthesized and functionalized palladium/platinum nanoparticles (Pd/Pt NPs) with excellent catalytic activity with anti-*Staphylococcus aureus* antibodies to construct a “visual–colorimetric–photothermal” multimodal lateral flow immunoassay (LFIA) for the sensitive detection of *Staphylococcus aureus*, addressing the increasing demand for point-of-care testing (POCT) of bacterial pathogens in field settings. Using a sandwich immunoassay format, Pd/Pt@Ab1 nanoparticles were specifically captured on the test (T) line, enabling visual immunocatalytic signal generation. *Staphylococcus aureus* was detected not only through the colorimetric response resulting from the Pd/Pt@Ab1 NPs-catalyzed oxidation of TMB, but also via the photothermal properties of oxidized TMB (oxTMB) under 808 nm laser excitation, which also allows for colorimetric signal readout. This approach established a multimodal sensing platform that integrates visual, colorimetric, and photothermal detection modalities. The platform exhibited an ultralow limit of detection (LOD) of 4 CFU/mL, with a wide dynamic range spanning from 10^2^ to 10^7^ CFU/mL. The integration of diverse, complementary, and synergistic signal outputs significantly enhanced the flexibility, practicality, and accuracy of the assay, demonstrating excellent potential as a versatile point-of-care testing (POCT) platform for application in a broad range of diagnostic scenarios (Figure 12).

**Table 2:** The application of nanozymes in POCT.

Sensing Methods	Detected Substances	Detection Limits	Linear Range	References
Colorimetric	Human Hemoglobin	1 ng·mL^−1^	–	[87]
	SARS-CoV-2	200 copies·mL^−1^	–	[88]
	Glucose	3.125 μM	10–900 μM	[89]
	Influenza Viruses	5.0 × 10^−12^ g·mL^−1^	5.0 × 10^−15^–5.0 × 10^−6^ g·mL^−1^	[91]
Electrochemical	H_2_O_2_	1.62 μM	1 μM–3 mM	[96]
Fluorescent	Uric Acid	24 nM	–	[101]
	Captopril	0.45 μM	0.5–30 μM	[102]
	*Escherichia coli*	1.74 cfu·mL^−1^	10–10^7^ cfu·mL^−1^	[103]
Chemiluminescence	SARS-CoV-2	0.1 ng·mL^−1^	0.2–100 ng·mL^−1^	[105]
	Epstein–Barr virus	–	–	[106]
SERS	Nitrite	1.67 nM	1–100 nM	[114]
Pressure	Carcinoembryonic Antigen	0.13 ng·mL^−1^	0.2–60 ng/mL	[118]
Chemiluminescence	Gentamicin	0.33 pg·mL^−1^	0.001–100 ng mL^−1^	[120]
photothermal	Gentamicin	16.67 pg/mL^−1^	0.05–100 ng mL^−1^	[120]
Colorimetric	Microcystin-LR	0.26 ng·mL^−1^	–	[121]
SERS	Microcystin-LR	0.032 ng·mL^−1^	–	[121]
Colorimetric	H_2_S	0.8 μM	–	[122]
Electrochemical	H_2_S	0.76 nM	–	[122]
Colorimetric	*Escherichia coli*	–	10^2^–10^6^ CFU mL^−1^	[123]
fluorescence	*Escherichia coli*	–	10^2^–10^6^ CFU mL^−1^	[123]
Colorimetric	Acetylcholinesterase	0.003 U L^−1^	–	[124]
Photothermal	Acetylcholinesterase	0.003 U L^−1^	–	[124]
Visual–colorimetric–photothermal LFIA	*Staphylococcus aureus*	4 CFU mL^−1^	10^2^–10^7^ CFU mL^−1^	[125]

Note: The detection line of commercially available colloidal gold strips is 100 ng/mL.

## 4. Nanozymes for POCT: Challenges and Perspectives

The emergence of nanozymes has established a bridge between inorganic materials and diverse fields such as biomedicine, agricultural and food sciences, and environmental remediation [126]. Currently, POCT platforms based on nanozyme sensing have evolved into an emerging detection technology across multiple disciplines. However, despite the close integration of nanozymes with contemporary POCT technologies—continuously providing impetus for their development-several challenges and issues persist. For instance, strategies for modulating nanozyme activity, elucidating catalytic mechanisms, achieving uniform dispersion, and scaling up production still require further in-depth investigation. Moreover, when applying nanozymes to POCT, researchers must confront and overcome a series of technical hurdles. Owing to the lack of clarity in the underlying mechanisms, the variety of nanozymes currently applicable to POCT is relatively limited, and each nanozyme exhibits inherent activity constraints. As a result, certain nanozyme-based POCT methods may present narrow detection and linear ranges, thereby falling short of clinical requirements for biomarkers at varying concentration levels and limiting their broader application. Furthermore, the rapid advancement of both nanozyme and POCT technologies has led to the absence of fully standardized technical protocols and regulations, which in turn impedes the practical application of research findings and the widespread adoption of these technologies.

### 4.1. Challenges Associated with Insufficient Catalytic Activity in Nanozymes

The distribution of active sites and catalytic mechanisms in nanozymes differs from those of natural enzymes, and the reported catalytic activities of most nanozymes are generally lower than those of natural enzymes, necessitating further investigation and enhancement of their catalytic performance. Fortunately, recent studies have demonstrated that the catalytic activity of nanozymes can now rival, and even surpass, that of natural enzymes. The active sites on the surface of nanozymes are critical to their enzyme-mimicking properties, and precise control over these sites can enhance the catalytic specificity toward particular substrates, which is beneficial for the accurate measurement of analytes in POCT [127]. For example, classical platinum (Pt) nanozymes possess excellent electron transfer capabilities, which can improve electron transport and thereby amplify electrochemical signals. However, the stability and selectivity of natural nanozyme-based sensing systems are easily influenced by factors such as pH, temperature, humidity, and the intrinsic structure of the nanozyme, which creates both challenges and opportunities for constructing ideal nanozyme sensors. Surface modification of nanozymes and the development of chiral nanozymes can enhance specific recognition; nevertheless, such modifications may negatively impact the catalytic efficiency. Balancing the catalytic activity and the biosensing specificity of nanozymes remains an ongoing challenge. While probing into the catalytic mechanisms and undertaking structural modifications of nanozymes, it is imperative to design more stable and highly efficient nanozymes to overcome these challenges.

### 4.2. Challenges in the Uniformity and Dispersibility of Nanozymes

The uniformity and dispersibility of nanozymes are intimately linked to their enzyme-mimetic catalytic activities, a feature that is critical for their application in POCT technologies. Uniformly dispersed nanozymes can maximize contact with substrates, minimize non-specific adsorption, and enhance catalytic efficiency, thereby improving detection sensitivity and ensuring the stability and reliability of test results. However, their dispersibility is highly susceptible to environmental factors such as temperature, humidity, and pH, and may deteriorate over long-term storage due to aggregation or oxidation. Currently, variability across different batches and synthesis conditions results in inconsistent performance in POCT, leading to fluctuations in detection outcomes. Moreover, the preparation processes for nanozymes lack standardized protocols; even minor variations in reaction conditions during common chemical synthesis methods can lead to a broader particle size distribution and irregular morphologies, ultimately compromising dispersibility. These challenges necessitate ongoing attention and continual refinement by researchers in the nanozyme field.

### 4.3. Challenges in the Mass Production of Nanozymes

The cost of mass-producing nanozymes remains relatively high, primarily due to the added expenses of the synthesis process and quality control. For instance, the costs associated with characterization tests can be significant, and the inherent uncontrollability of in-process characterization exacerbates quality differences. Studies have shown that variations in oxygen vacancy density among different batches from the same production line can lead to substantial differences in their enzyme-mimetic activities. Traditional bulk synthesis methods cannot precisely regulate the active sites of nanozymes, and the difficulty in controlling structural uniformity represents a major bottleneck in large-scale production. Moreover, aggregation of nanoparticles during production reduces their specific surface area, which can severely compromise their catalytic performance. Overcoming these engineering challenges not only helps address the limitations of nanozymes, but also expands their application potential in various fields, further driving multidisciplinary research and offering significant scientific value and broad industrial prospects.

### 4.4. Prospects for Nanozyme-Based POCT Technologies

Nanozyme-based POCT technologies have been extensively investigated. Future developments in this field are expected to focus on intelligence and multifunctional integration. For example, leveraging AI-based simulations of natural enzyme active centers will facilitate the rational design and development of nanozymes exhibiting enhanced catalytic activity and superior substrate specificity. Combining the precision imaging capabilities of smartphones, advanced image processing algorithms, and highly sensitive colorimetric detection with POCT can substantially enhance both convenience and efficiency, especially by expanding applications in resource-limited settings. Moreover, POCT sensor platforms based on smartphones miniaturize conventional bulky biochemical analyzers and incorporate wireless data transmission, enabling rapid and clear display of nanozyme-based colorimetric signals on a screen, while facilitating quantitative analysis of analytes through integrated data processing systems. Furthermore, the latest microfluidic sensors have achieved a highly integrated chip design that combines high performance, low detection limits, miniaturization, and controllability. Microfluidic technology enables the handling of small-volume samples, rapid sample injection, mixing, and reaction within a compact chip. Such microfluidic chips exhibit tremendous potential in POCT systems by integrating ultra-low detection limits (LOD < 1 nM), precise fluid control, and multiple reaction chambers. They achieve rapid detection (in less than 15 min) while optimizing reagent consumption (≤50 μL), marking a significant step toward deployable on-site diagnostic solutions. Innovative POCT technologies continue to emerge. In the future, the deep integration of artificial intelligence (AI) with POCT is anticipated to become an extremely promising and indispensable trend. Machine learning-enhanced pattern recognition algorithms have demonstrated accuracies of 92–97% in automated result interpretation, significantly outperforming traditional analytical methods. Overall, the vast prospects of combining nanozymes with POCT warrant our utmost expectation and call for dedicated research efforts.

## 5. Conclusions

In summary, nanozyme-driven on-site detection has made remarkable strides in multimodal sensing and the analysis of clinical and environmental specimens. By mimicking diverse natural enzymes and employing particle-size tuning, atomic doping, and biomimetic active-site engineering, nanozymes achieve substantially enhanced catalytic efficiencies. Moreover, point-of-care testing (POCT) platforms based on nanozymes have expanded from simple colorimetric assays to electrochemical, fluorescence, chemiluminescence, SERS, and even integrated multimodal readouts.

Nonetheless, several pivotal challenges remain: the catalytic activity of nanozymes is still inferior to that of natural enzymes, large-scale synthesis is hampered by scalability issues and pronounced batch-to-batch variability, and standardized production protocols are largely absent. To overcome these barriers, it is essential to integrate atomic-level mechanistic investigations and biomimetic design with machine learning-guided materials discovery; to apply controlled polymerization or surface-coating strategies to enhance both stability and biocompatibility; to implement continuous-flow synthesis coupled with in-line characterization for high-throughput, cost-effective manufacture; and to fully merge microfluidic architectures with AI-driven image analysis and smart-device interfaces. Such a comprehensive strategy will expedite the industrial translation of nanozyme-based point-of-care testing (POCT), establishing these materials as cornerstone technologies for next-generation on-site diagnostics in precision medicine, environmental monitoring, and food safety.
